# Effects of Surface Chemistry Interaction on Primary Neural Stem Cell Neurosphere Responses

**DOI:** 10.1021/acsomega.1c02796

**Published:** 2021-07-19

**Authors:** Georghios Joseph, Rowan P. Orme, Theocharis Kyriacou, Rosemary A. Fricker, Paul Roach

**Affiliations:** † Institute for Science and Technology in Medicine, and School of Medicine, Keele University, Keele, Staffs ST5 5BG, U.K.; ‡ School of Computing and Mathematics, Keele University, Keele, Staffs ST5 5BG, U.K.; § Department of Chemistry, School of Science, Loughborough University, Loughborough, Leicestershire LE11 3TU, U.K.

## Abstract

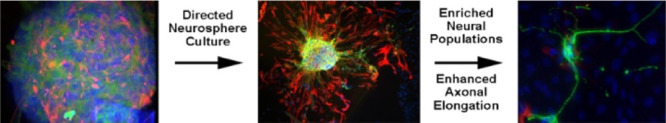

The characteristics of a material’s surface are extremely important when considering their interactions with biological species. Despite surface chemistry playing a critical role in mediating the responses of cells, there remains no single rule which dictates absolute performance; this is particularly challenging when considering the response of differing cell types to a range of materials. Here, we highlight the functional behavior of neural stem cells presented as neurospheres, with respect to a range of alkane-based self-assembled monolayers presenting different functional groups: OH, CO_2_H, NH_2_, phenyl, CH_3_, SH, and laminin. The influence of chemical cues was examined in terms of neurosphere spreading on each of these defined surfaces (cell adhesion and migration capacity) and neuronal versus glial marker expression. Measurements were made over a time series of 3, 5, and 7 days, showing a dynamic nature to the initial responses observed after seeding. While OH surfaces presented an excellent platform for glial migration, larger proportions of cells expressing neuronal β_3_-tubulin were found on SH- and laminin-coated surfaces. Axonal elongation was found to be initially similar on all surfaces with neurite lengths having a wider spread predominantly on NH_2_- and laminin-presenting surfaces. A generalized trend could not be found to correlate cellular responses with surface wettability, lipophilicity (log *P*), or charge/ionizability (p*K*_a_). These results highlight the potential for chemical cues to direct primary neural stem cell responses in contact with the defined materials. New biomaterials which control specific cell culture characteristics *in vitro* will streamline the up-scale manufacture of cellular therapies, with the enrichment of the required populations resulting from a defined material interaction.

## 1 Introduction

The manufacture of cells at scale is of great importance in the advancing of fields of regenerative medicine and tissue engineering. The large-scale manufacture of cells required to make significant availability of cell therapies is still very much limited by the costs and reproducibility of manufacturing.^1^ Many researchers are interested in better understanding the fundamental interactions of cells with their biomaterial surroundings *in vitro* in order to inform material design. Further, following the clinical application of cellular therapies in a host environment, there is little to no “control” of their responses; they require continued instruction/direction in order for the developing tissue to present the required cell type(s), architecture, and function. This is particularly important in complex tissues such as within the neural niche.^2^ Specific cell–biomaterial interactions are of absolute value to inform advanced biomaterial development, with surface chemistry playing a major role in the initial interactions and ensuing cell responses.

Chemical and physical properties of cell culture materials have long been studied in order to better impart control over tissue development,^3,4^ although it remains conventional, within neuronal culture protocols, to use proteins as a biomimic of the laminin-rich developmental niche.^5^ Electrostatic charges between cell membranes and substrates have been used to describe some cell adhesion behaviors, although surface wettability has remained a critical parameter for discussion among the biomaterial community. It is thought that laminin enhances neural cell attachment initially through the presentation of a positive charge, followed by selective integrin binding.^6^ Other biological mimics have been studied for the expansion of neural stem cells (NSCs),^7^ although there is a specific limitation for high throughput governed by the lack of chemical understanding at such biointerfaces.

Efforts to study cell responses on defined chemical surfaces often describe observations in relation to the specific chemical moiety, offering benefits over other umbrella terms as more information is provided about the actual chemical properties rather than a universal assessment of, for example, wettability.^8^ A more global assessment of surface chemistry can be achieved by taking into account all of the above factors, using a partition coefficient (log *P*) derived from the untethered molecule.^9^ The high-throughput assessment of cell–surface responses has expanded rapidly with microdot printing and biostatistics approaches, with efforts particularly focused on how chemical functionality imparts particular cell characteristics.^10^

The controlled differentiation of NSCs has been a challenge, with many exquisite strategies being developed relying on transient biochemical triggers with a plethora of expensive bioinstructive supplements to mimic the neural niche environment.^11^ Hierarchical topographical patterning has been utilized to enhance NSC differentiation,^12^ while others have investigated the potential for surface chemical agents to be used to control the adhesion and subsequent morphological and phenotypical changes in NSCs.^13^ Studying a range of chemical functionalities in terms of the head group, it was suggested that NSCs cultured in serum-free conditions tended toward oligodendrocytes on SO_3_H-terminated surfaces, with NH_2_ surfaces generally presenting higher proportions of neuronal cells. Here, the capacity of cells to migrate from adhering neurospheres was thought to be indicative of the differentiation potential. Hung et al. have likewise demonstrated the potential for patterning of substrate biochemistry in driving stem cell responses.^14^ Dopamine was patterned on substrates to enhance the guided differentiation of mesenchymal stem cells toward neural and endothelial lineages. Here, we report the study of primary NSC interaction with defined surface chemical groups in serum conditions, taking into account various aspects of global surface chemical properties and cell responses, particularly fractional populations of neurons versus glia derived from NSCs/progenitors, as well as axonal lengths, morphological, and migration assessments. Responses of neurospheres (as opposed to single cells) are important to better understand NSCs within a biological niche and how they can be affected, or indeed directed, by external factors.^15^ Neurosphere models are well used, being particularly important when considering developing *in vitro* studies and biomaterial design. Defined alkane self-assembled monolayers (SAMs) were prepared to present a range of head groups with varying chemical characteristics: amine (NH_2_), carboxyl (CO_2_H), methyl (CH_3_), phenyl (Ph), and thiol (SH) alongside a hydroxyl (OH) surface, which could be considered as a control as a “bare” glass surface. The responses of primary NSCs, seeded as neurospheres onto these surfaces, are discussed to highlight relationships with respect to quantifiable surface chemical properties. The neurospheres were allowed to freely interact with the substrate, being observed across a large surface area; responses are observed due to the differences in the presented chemical groups at the surface with any heterogeneity arising from this interaction and subsequent cellular maturation. Although there are a plethora of methods to assess such a response, here we examined the expression for the well-understood glial-derived growth factor (GFAP) and β_3_-tubulin markers, as well as the neurosphere-spreading area and neurite length characteristics. Through better understanding and then controlling cell–material interactions, we aim to direct cellular processes and advance our capabilities in terms of cell product homogeneity and reproducibility.

## 2 Results and Discussion

### 2.1 Culture Environment

Silane SAMs were produced presenting a range of terminal functional groups. All surfaces were characterized by drop shape analysis (DSA, Supporting Information, Figure S1) and X-ray photoelectron spectroscopy (XPS, Supporting Information, Figure S2) to confirm the presence of the SAM modification. These complimentary techniques demonstrate that the glass substrates used were modified as expected, with wettabilities being in the range expected for modified “smooth” glass coverslips, as shown in Table 1. Water contact angles for all surfaces presented within this study are reproducible, and are in good correspondence with those expected, bearing in mind that the terminal head chemical functionality sits directly atop a hydrocarbon chain which is known to contribute to the observed contact angle.^19^

**Table 1:** Surface Chemical Characteristics; Wettability SD Given as *n* = 12^a^

functionality	WCA/° ± SD	*A* log *P*	p*K*_a_
–CO_2_H	24.9 ± 2.2	–0.16	2.00
–NH_2_	29.2 ± 6.6	0.42	10.71^25^
–SH	45.6 ± 8.2	1.73	11.94^26^
–OH	32.1 ± 7.4	0.65	4.9^27^
–Ph	64.2 ± 2.6	2.84	43.00^27^
–CH_3_	65.3 ± 3.5	0.78	48.00^27^
laminin	60.4 ± 7.2	N/A	pI ∼ 5

a Values obtained *via* modeling being supported (where presented) by the literature.

Surfaces were assessed to be chemically stable in aqueous conditions for at least up to 3 weeks (data not presented). Although it is well understood that proteins and other biological molecules will adsorb rapidly to the surface, changing with respect to individual protein conformation and overall surface protein composition,^20^ the study here used serum-supplemented media, although it is acknowledged that other researchers may use serum-free media supplemented with N-2, B27, or other reagents. The adsorbed protein later is known to play a role in neural cell interaction,^21^ with the serum being important for the attachment of neurospheres to biomaterials as shown by Hung et al.^22^ Here, polymeric materials were compared in serum-containing media to better understand how the serum-conditioning characteristics affected the development of cells derived from neurospheres.

Serum-free media are widely used for the neurosphere culture, although each and every one of these ranges in the supplements used in order to control neurosphere development, albeit that much of the propagation of change induced depends on initial interactions with substrates. A whole host of different growth factors and small molecules can be added as supplements, including BMP2,^22^ retinoic acid, and neurotrophins,^23^ basic fibroblast growth factor (bFGF), and epidermal growth factor^24^ to name a few.

The current study used serum-rich media, which is in line with other studies wanting to reduce the variability introduced by selecting any one supplement over another and also wanting to examine the fundamental effects of surface chemistry potential on NSC responses within close to biological niche conditions. The use of serum-rich media ensures that a plethora of proteins are presented, minimizing any sample–sample variation and indeed any variance observed across the surface between individual neurospheres. The concentration of any signaling factors released by cells is reduced to insignificance, with only the surface chemical changes being apparent as a difference presented to neurospheres in different samples. The only variable between the samples was the chemistry presented by the substrate.

### 2.2 Neurosphere Spreading

The neurosphere response to surface chemistry was investigated by fluorescence microscopy using markers to identify glial and neuronal cell populations. This was a visual qualitative measure initially with clear differences observed with the neurospheres interacting across the range of surface chemistries; quantitative information was obtained by measuring the neurosphere area and how this evolved over time. The neurospheres were observed to start attaching to surfaces during the first few hours of incubation, with those cultured above more hydrophobic surfaces generally taking longer to attach. A two-way ANOVA showed that a highly significant difference (*p* < 10^–7^) between the neurosphere-spreading areas was observed on all the surfaces tested, highlighting the impact of surface chemistry on the neurosphere–surface interaction.

**Figure 1:**
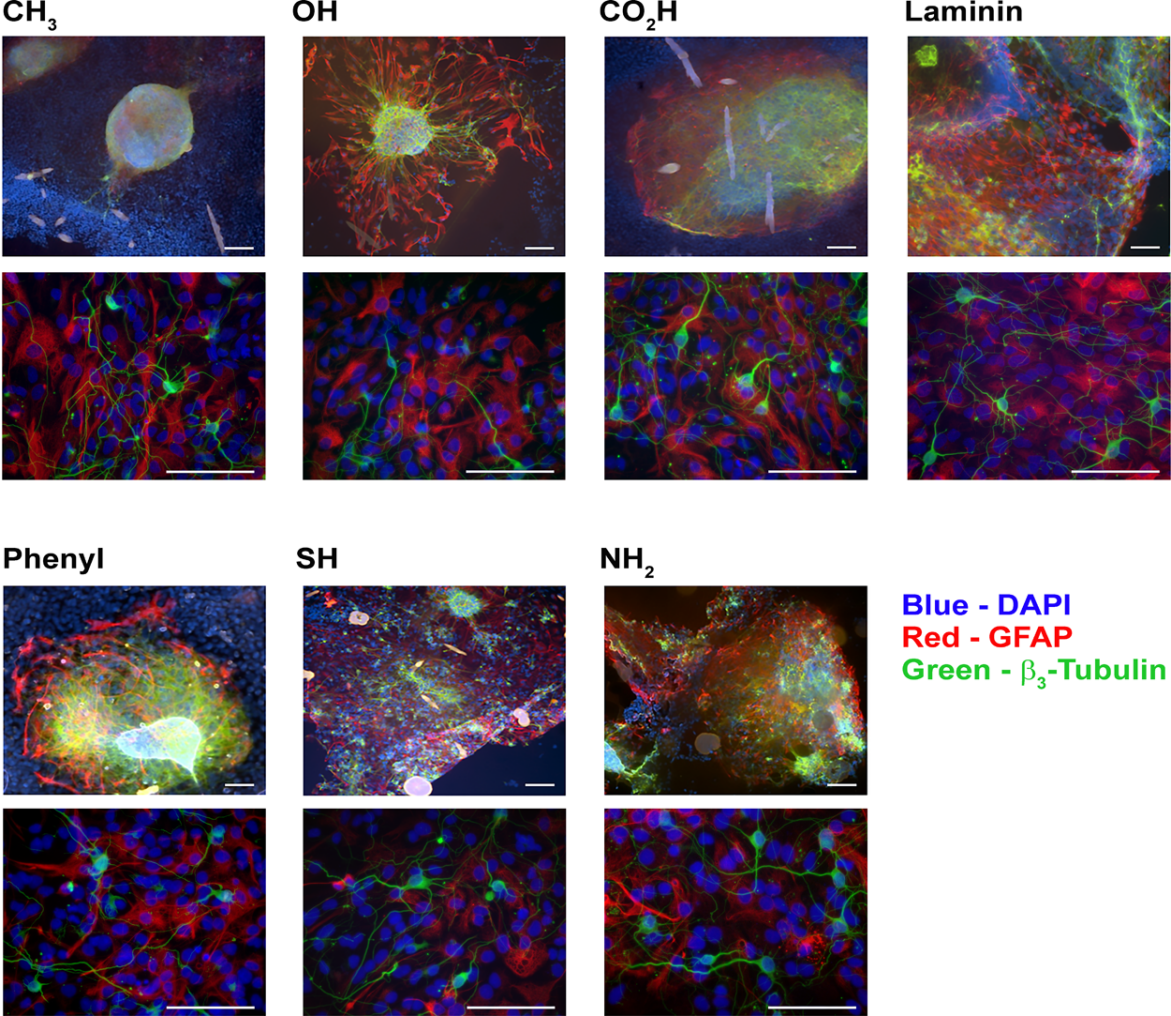
Images of neurospheres attached to a range of defined surface chemistries using fluorescence microscopy. Top image for each panel shows representative images for neurospheres, with the lower image showing a higher resolution detail for individual cells from which cell counts and neurite lengths were measured. Cells shown after 3 days in culture. Scale bars 100 μm.

**Figure 2:**
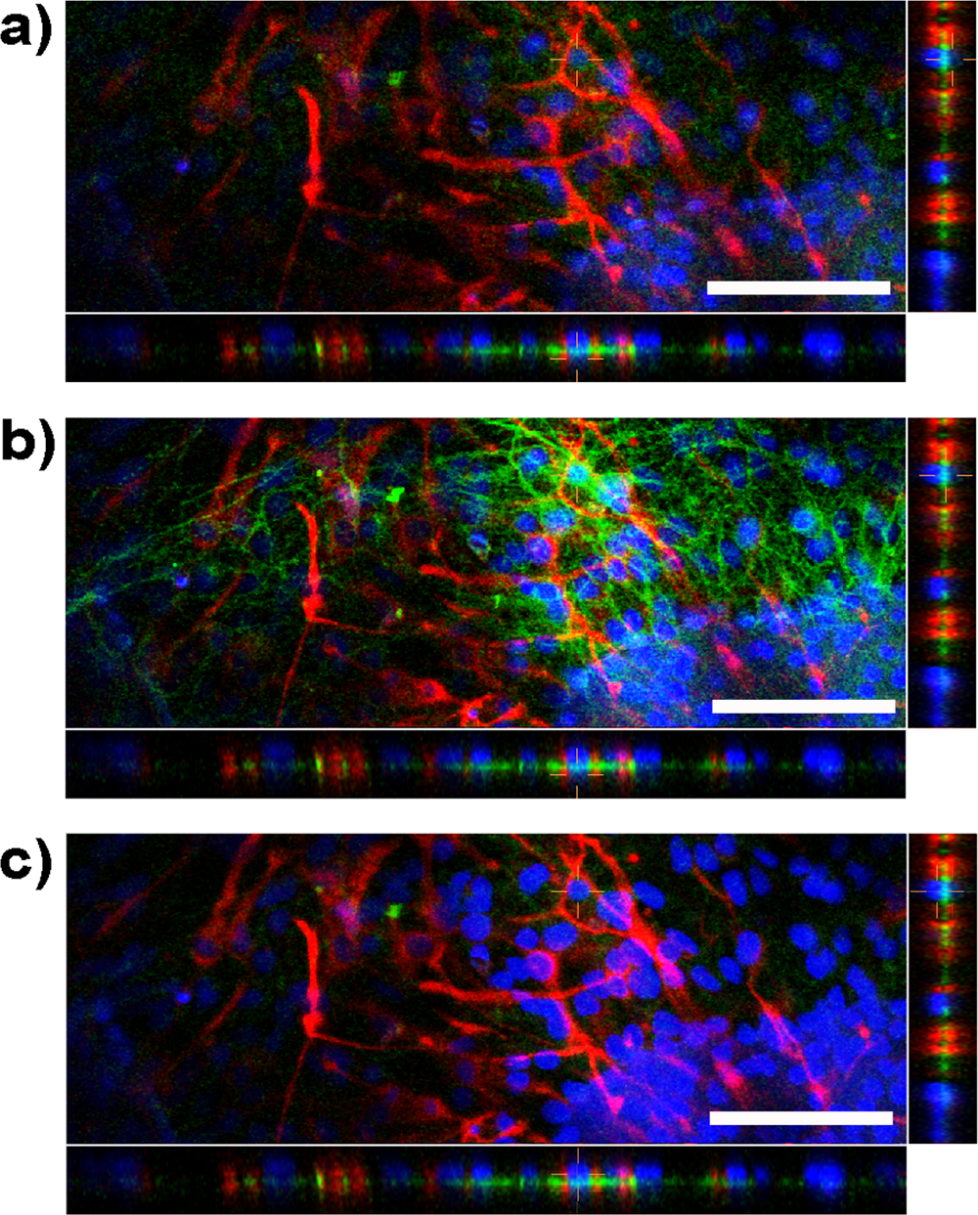
Confocal image of a neurosphere section cultured on a hydroxyl surface for 3 days. Layer slices shown are (a) lower, (b) middle, and (c) upper. Stains are as follows: GFAP (red), β_3_-tubulin (green), and DAPI (blue). Scale bars indicate 100 μm; each optical slice was 45 μm thick.

Neurospheres were found to attach to all the SAM-modified substrates after 4 h, although generally there was a more rapid attachment and spreading onto surfaces presenting hydrophilic chemical groups, as shown in Figure 1. In general, populations migrating away from the neurosphere edges were found to stain positive for either glial (GFAP) or neuronal (β_3_-tubulin) markers. The underlying surface of the neurospheres was found to present a glial bed, with a mixture of neuronal and glial cells within the neurosphere body (Figure 2). In some cell preparations, there are clearly cells outside of the neurosphere boundary, staining negative for GFAP and β_3_-tubulin, these can be assumed to be seeding in addition to the neurospheres, possibly resulting as a small contamination of fibroblastic cells originating from the meninges, as shown in Figure 1.

The results demonstrate low variability between population samples and neurosphere–neurosphere on each of the samples. This indicates that although the additional cells are present, they do not have a significant effect on the conditions. Cells were dissected and expanded in batches, being plated as a population across all surfaces from the same batch. This reduced any variability across the samples to only change the chemistry presented by the substrate—all cell populations were consistent.

Glial cells were observed to migrate further from the neurosphere boundary, particularly highlighted on the hydroxyl surface, providing a bed on which the neurons were attached. The migration of cells was quantified in terms of neurosphere spreading, taking into account the differing interactions of the cells within the neurospheres, interacting more strongly with some surfaces presented as an increase in the spreading area. The presentation of the neurospheres depended greatly upon the interaction with differing surface chemical groups, with a proportion of each cell being found to differ in their distribution across the range of surfaces tested, highlighting the initial surface-driven differentiation response (Figure 1).

**Figure 3:**
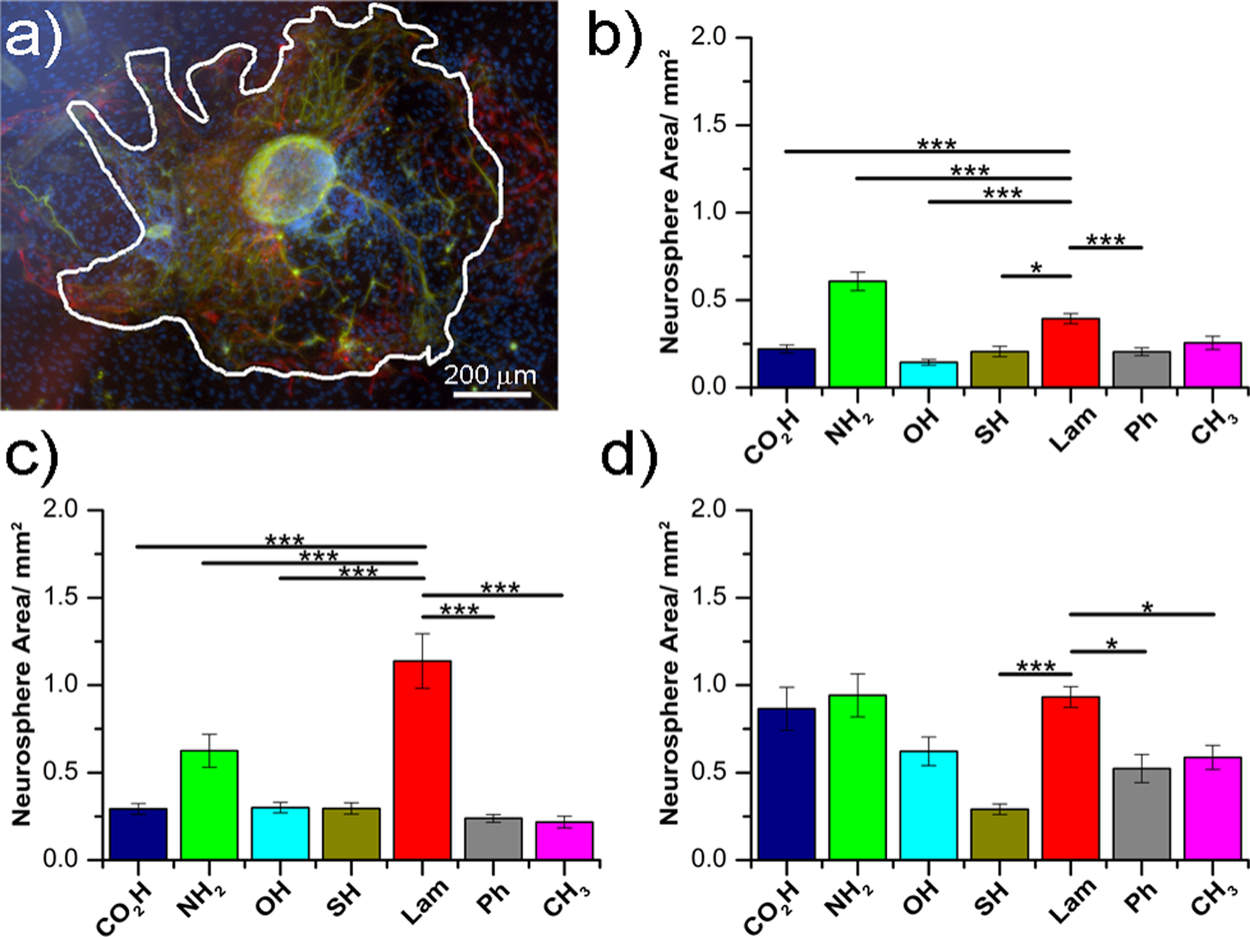
Neurosphere spreading capacity on different surface chemistries; (a) fluorescence microscopy image of neurospheres on a hydroxyl-functionalized surface at day 3 depicting the area boundary as a white line (green—β_3_-tubulin, red—GFAP, and blue—DAPI nuclear stain); plots show area measurements after (b) 3 days, (c) 5 days, and (d) 7 days culture. Error bars present ± standard deviation. Statistics are only shown comparing control laminin-coated surfaces: *p ≤ 0.05, **p ≤ 0.01, ***p ≤ 0.001. Surface functionalities are ordered in decreasing wettability, left to right.

After 3 days in culture (3 days postseeding of the neurospheres onto their respective surfaces), there was a low variation in neurosphere spreading and degree of cell migration from their parent neurosphere on individual surfaces (i.e., deviation among sample repeats was low; Figure 3).

However, distinct spreading patterns of neurons and glia from the spheres were observed by fluorescence microscopy, being dependent upon the surface functionality. Amine-terminated surfaces gave rise to the largest spreading neurospheres (0.61 ± 0.05 mm^2^), being larger than those on laminin surfaces (0.39 ± 0.03 mm^2^). All other surfaces were similar to each other, with the lowest spreading capacity presented by hydroxyl SAMs (0.14 ± 0.02 mm^2^). Differences observed in neurosphere spreading across the range of surfaces tested did not appear to follow a direct trend with respect to surface wettability. A marked increase in the neurosphere spreading was observed on laminin after 5 days in culture (1.14 ± 0.16 mm^2^) and hydroxyl (0.30 ± 0.03 mm^2^)-coated surfaces on day 5 compared with day 3, and other surfaces were showing either a slight or no increase in the neurosphere spreading area.

A significant increase in the neurosphere spreading area was observed when comparing day 7 to day 3 data from almost all the surfaces tested (Figure 3). Neurospheres cultured on thiolated surfaces were the exception, showing no significant increase in spreading over the whole culture period.

### 2.3 Density of Neurons

**Figure 4:**
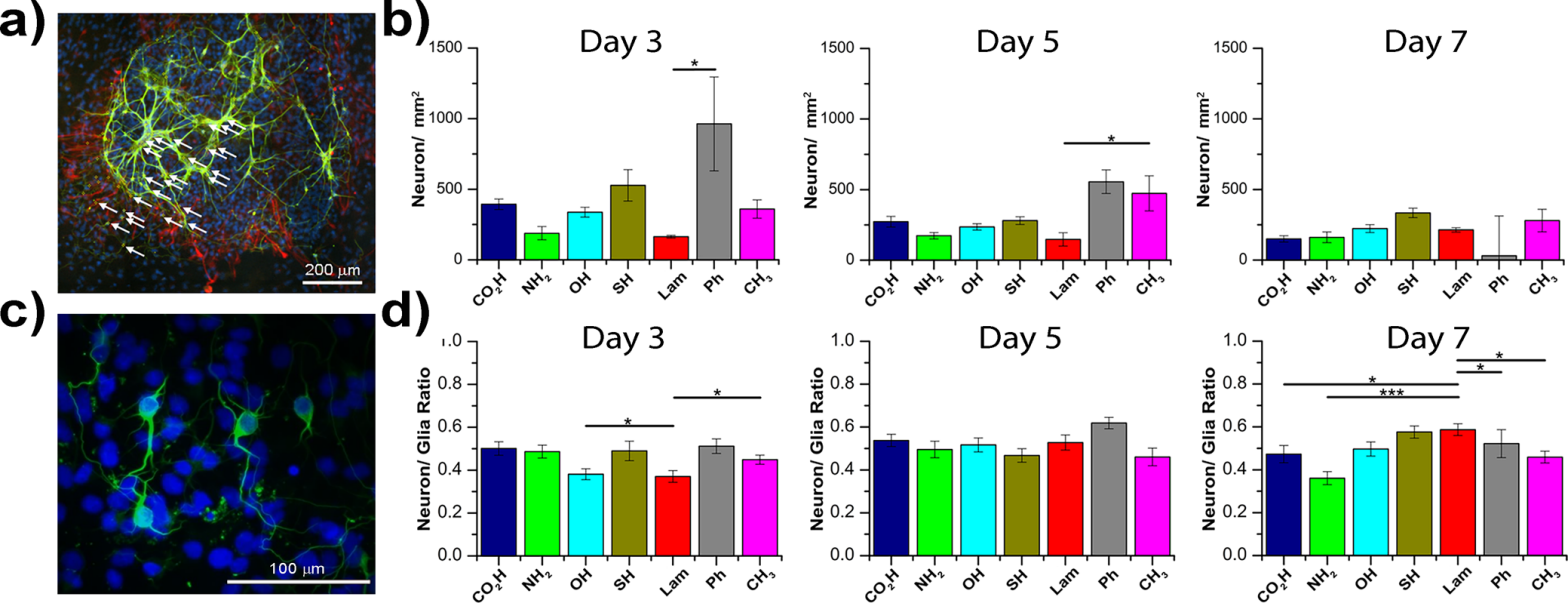
Neuronal and neuron/glia ratios presented over time of culture: (a,c) representative images of cells on day 5 of culture on amine and hydroxyl surfaces. Staining represents β_3_-tubulin (green), GFAP (red), and DAPI (blue) with white arrows indicating neurons in (a). Plots show measurements of neuron densities (b) and neurons/glia ratio (d) as a function of surface area. Error bars present ± standard deviation. Statistics are only shown comparing to control laminin-coated surfaces for clarity: *p ≤ 0.05, **p ≤ 0.01, ***p ≤ 0.001.

Cells across the neurosphere area were counted as a means of quantifying the capacity of neural progenitors to be steered toward neuronal lineage. Measurements were taken over several days of culture, with neurospheres becoming flattened enough for reliable measurements, that is, no cells were obscured by the depth of the neurosphere mass (Figure 4a).

The quantification of neuron numbers was normalized to the surface area on which they resided due to differences in initial neurosphere size and cell numbers. Two-way ANOVA analysis conducted over the three repeat samples for the three repeat tissue collections (i.e., nine samples overall) indicated significant differences between population means of all surfaces (*p* < 0.001) and at all time-points tested (*p* < 0.001). The interaction between the two factors was not significant. A Tukey’s *post hoc* test was also performed to assess individual differences between test populations, showing significantly higher density only on phenyl surfaces.

Neuron densities were found to be generally similar on individual surfaces tested at day 3 with some variance being observed across cell densities counted on phenyl- and thiol-terminated surfaces. This demonstrates overall reproducibility. Most surfaces after 3 days in culture presented a similar density of neurons; phenyl surfaces were the only substrate to show a significantly higher density (Figure 4b). Cell densities were found to be ∼500 neurons mm^–2^, with a highly significant difference observed between laminin (∼160 ± 10 mm^–2^) and phenyl (∼960 ± 330 mm^–2^).

By day 5, little difference was found between replicates on each surface. Significant differences were found (at a level *p* < 0.05) when comparing surfaces to phenyl, with the exception between phenyl and methyl surfaces, which were not significantly different. This indicates that there may be some correlation between surface wettability and density of neurons, although certainly this method of characterization is not encompassing for all responses observed.

Less change between the surfaces was observed for neuron densities measured at 7 days, with significant differences only being observed when comparing measurements made on carboxyl and amine surfaces to surfaces presenting thiol termination. Importantly, neuron densities were found not to decrease significantly over time, even though neurosphere areas increased. The only surface on which a decrease in cell density was observed was that with the phenyl terminal chemistry.

### 2.4 Neuronal Versus Glial Cell Populations

Although the regeneration of electrically functional neural tissue requires high numbers of neurons, supporting glial cells are often found to dominate cultures due to their proliferation. The normalization of neuronal densities to account for the total cell numbers afforded from neurospheres (neurons and glia) served as a better indicator for differences in cell–surface responses. On all the surfaces tested, a mixture of neuron and glial cells is observed, as shown in Figure 4. Two-way ANOVA analysis showed significant differences between population means of all the surfaces tested at all time points (*p* < 0.05).

Neuron to glia ratios at day 3 indicated a higher proportion of glia present on all surfaces, with neuron/glia ratios ranging from ∼0.36 to 0.52, as shown in Figure 4d. Lower proportions of neurons were observed on hydroxyl- and laminin-coated substrates compared to all the others tested. At day 5, similar ratios were observed, with fewer differences found from surface to surface. Both hydroxyl and laminin presented population ratios much closer to other surfaces at ∼0.52. Only neuron/glia ratios presented on thiol and phenyl surfaces were significantly different at a level of *p* < 0.05, with the highest neuron populations being observed on phenyl surfaces. The highest fraction of neurons was observed by day 7, with laminin presenting significantly greater neuron fractions compared to other surfaces.

### 2.5 Neurite Length

**Figure 5:**
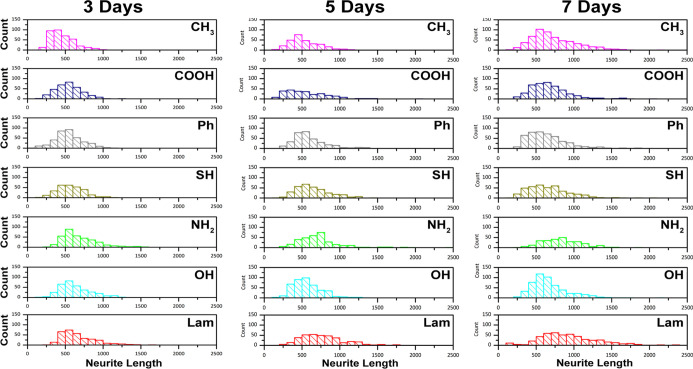
Neurite length distributions measured on surfaces presenting differing surface chemistries. Data collected from fluorescence microscopy images at varying time points.

Regeneration of nerve tissue relies heavily on the ability of neuronal projections to effectively communicate to neighboring cells, so that electrical conduction across large sections of the tissue can be established. Neurite lengthening permitted by the material is a key indicator of this *in vitro*. Neurites were measured at ∼300 neurons per surface, taking only those cells where β_3_-tubulin clearly defines the entire neurite length. From each surface, a distribution of lengths was obtained, as would be expected due to the differing time of individual cell–surface interaction. These are presented as histograms allowing a direct comparison between surfaces at varying time points (Figure 5).

After 3 days in culture, neurite length distributions were found to be the highest on laminin surfaces, with the smallest length distribution obtained on methyl-terminated surfaces. Methyl surfaces also produced the largest number of shorter neurites compared to other surfaces. Neurite length distribution was not found to significantly increase by day 5, with similar trends between substrates observed in those at day 3. A much tighter distribution was, however, observed in hydroxyl surfaces, with the distribution being smaller between the two time points. The largest neurites were observed on the amine and laminin surfaces, being ∼1000 μm compared to those on the other surfaces reaching average lengths of ∼650 μm. Laminin surfaces presented the broadest spectrum of neurite lengths after 7 days.

## 3 Discussion

### 3.1 Consideration of Surface Characteristics

Although surface chemistry has been widely investigated in terms of directing cell responses, there are no overarching correlations between the chemical property of the surface and its impact on biological response. Functional head groups presented at the surface are often used as a measure of change at the surface, for example, amine versus carboxylic acid surfaces. Although these labels allow the comparison of substrates, it is acknowledged that they are far from appropriate for the full characterization. In general, both amine- and carboxyl-terminated surfaces have been used to provide a mimic of biological surfaces, and therefore are generally known to support cell adhesion and spreading.^28^

In the present study, a selection of defined SAMs were fabricated such that a comprehensive investigation of surface chemical parameters could be assessed with respect to their ability to impact on NSCs in the form of neurospheres. Methyl (CH_3_)-, phenyl (Ph)-, amino (NH_2_)-, hydroxyl (OH)-, carboxyl (CO_2_H)-, and thiol (SH)- functionalized silanes were fabricated with a direct comparison against the laminin-modified glass substrate, which is the current “gold standard” onto which neural cells adhere and spread well.

### 3.2 Neural Cell–Surface Interaction

Neurospheres cultured on the range of surfaces tested showed very different characteristics, which evolved differently over the 7 days of culture. We demonstrate the potential to infer an increase in the capacity of neuron differentiation, along with differences in neurite elongation responses. Upon the interaction with surfaces, cells within neurospheres were found to differentiate forming neurons and glia (GFAP or β_3_-tubulin positive); these migrated away from the neurosphere at different rates depending on the initial cell–substrate interaction (Figure 1). Others have previously demonstrated the capacity for neurosphere differentiation, with this process being highly dependent upon the time of maturation.^15^ In the present study, glia were found to spread well on all the surfaces, while neurons remain within the body of the sphere on methyl and hydroxyl surfaces, being supported on a glial underlayer (Figure 2). Silane surfaces have previously been studied by Ren et al., where the authors observed a cortical-derived neurosphere response to surface functionalization.^13^ The neural specific marker β_3_-tubulin was expressed at higher levels on carboxyl compared to amine surfaces, indicating a degree of control over NSC differentiation.

#### 3.2.1 Neurosphere Spreading

*In vivo* and *in vitro* the neural stem and progenitor cells reside in distinct niches,^13^ which maintain self-renewal, division, and differentiation because the niche provides a good environment for cell-to-cell signaling and region specific signaling.^29^ As the neural stem and precursor cells develop into mature postmitotic neurons, their density decreases substantially to develop adult tissues and structures.^30^ A low neural density is therefore considered an indicator of tissue maturation. This process is driven by the interaction of cells with “adhesive” environments, allowing differentiation and, in the case of neurospheres, attachment to surfaces *in vitro*, driving a homeotypic short-range migration *en masse* out of the neurosphere body.^31^

In the present study, the spreading capacity of neurospheres was measured as a function of the surface area coverage, (Figure 3). While laminin-coated surfaces acted as a positive control, enabling good neurosphere adhesion and spreading, phenyl and methyl surfaces were generally observed to hinder initial cell migration. Amine surfaces, commonly suggested to be chemically similar to laminin due to the presence of many multiple primary amine containing residues along its backbone, also enabled high levels of spreading. After 3 days in culture, amine surfaces in fact showed a significantly higher average surface area of neurosphere contact compared to those on laminin surfaces, although no major differences were observed after 7 days. For these surfaces, a rapid migration of glia was observed away from the neurosphere body at day 3, with subsequent neuron migration observed at day 5. Mats of mixed cell populations were formed, giving a higher area of cell spreading than all other surfaces because neurons tend to migrate on a glia bed.^15^

Thiol-, phenyl-, and carboxyl-terminated surfaces showed comparable responses, although cell migration occurred somewhat slower to those observed on amine and laminin surfaces. Carboxyl and thiol surfaces produced cell mats to a lesser extent compared to amine surfaces, housing mixed populations of neurons and glia. Negatively charged carboxyl surfaces showed similar trends in terms of neuron/glia ratios, while the measured densities of neurons were found to decrease significantly over time. The migration of neurons from the neurosphere body gives rise to much larger spreading areas and therefore lower neuronal densities in these regions. This finding demonstrates the enhanced migration of neurons on the carboxyl surface compared to amine and laminin, suggesting a weaker attachment to this surface. All surfaces gave rise to an increase in neurosphere spreading by 7 days, except thiol-terminated surfaces. This was somewhat unexpected due to the nature of thiol to form disulfide bridges with proteins containing cysteine residues on their periphery, and thus form a bound protein, cell-mediating layer. It is possible that the thiolated surfaces promoted disulfide attachment to high abundant, non-adhesive proteins, for example, serum albumin, thus restricting the ability of cells to attach. It is interesting to note that although neurosphere adhesion initially occurred before 3 days in culture, spreading of neurospheres only took hold between 5 and 7 days on all surfaces except those terminated with amine or coated by laminin.

The data collected for neurosphere spreading show no correlation with regard to wettability at any of the time points analyzed. Surfaces classified either as hydrophilic or hydrophobic present with low spreading (CO_2_H vs CH_3_), as well as surfaces of mid-ranging wettability (SH) (Table 1). This suggests that chemical functionality plays an important role, further to denoting the surface wettability. Possibly, the largest of the umbrella terms used to define surface characteristics is wettability, being a generalized term that does not fully characterize the surface presented. As an example, many researchers will report the use of specific functionality-terminated surfaces without giving details about the molecule presenting this functionality; with only a proportion of surface-tethered molecules contributing to the observed contact angles, the upper presenting part of the molecule (4–5 carbon bond lengths deep) gives rise to wettability characteristics.^32^ The surface charge is also often discussed with respect to cell adhesion in terms of the associated surface charge accommodating that of the cell membrane. p*K*_a_ values can be used as an indicative measure of chemical charge as they represent equilibrium constants for molecular ionization. Laminin-coated surfaces could not be assigned a p*K*_a_ value due to the complexity of such a large molecule possessing many ionizable groups within its structure, although it has an apparent pI ∼5, suggesting a net negative charge at pH 7.4.

No correlation was observed between surface chemical p*K*_a_ and neurosphere surface area for 3 and 5 days. After 7 days in culture, a trend toward a higher neurosphere spreading was observed, with both high and low p*K*_a_, suggesting that both positive and negative surface charges play an important role in determining the cell–surface interaction. Less wettable surfaces (Ph and CH_3_) also supported neurosphere spreading, with less spreading being observed for mid-ranging SH presenting surfaces.

All cell culture was carried out under buffered conditions at pH 7.4, meaning that all surfaces excluding those presenting carboxyl termini were protonated. Both charged and non-wettable surfaces are known to drive protein adsorption through different processes: charge stabilization and hydrophobic interaction.^33^ Our results highlight surface polarity and charge to be the dominant factors affecting neural cell interaction, strongly suggesting that the protein layer adsorbed at the surface plays a key role in mediating cell attachment and subsequent responses.

The spreading capability of neurospheres was directly related to the ability of neurons and/or glia to migrate away from the body of the neurosphere. It is therefore very useful to look at the migration capacity of each of these populations to further understand the biological processes occurring. Cell population densities were normalized to the surface area to negate any changes related to increasing the number of cells possible to count as they migrated out of the neurosphere body. As spreading on laminin surfaces was observed to increase with increasing culture time but neuronal density remained relatively constant, it must be assumed that the number of neurons migrating out of the neurosphere also increased over time. This suggests either:

(1) longevity of the differentiation process with naïve cells coming into contact with the surface over the 7 days, migrating out of the neurosphere body before differentiating into neurons, or
(2) initial cell–surface interaction from within the body of the neurosphere initiates differentiation, and these more mature cells reorient within the neurosphere before migrating later.^31^

#### 3.2.2 Differentiation Potential

Materials play an important role in stem cell fate decisions. A key aspect of cell fate decisions are intrinsic and extrinsic signals. Neurospheres are tri-potent mixed cell spheroids of NSCs, glia, and neural progenitors;^34^ therefore, a useful property of functionalized surfaces would be to influence the NSC fate decisions. Neural and glia progenitors arise from NSCs through a process of symmetric and asymmetric divisions.^34^ Through temporal modulation notch signaling means commitment can be influenced toward glia or neuron phenotype.^35^ Transcription factors, such as the STAT3 pathway activation, have been shown to control NSC differentiation either toward neuron or glia lineage.^36^ Complex association of factors affecting gene switching, and therefore protein production, is often difficult to control within a mixture of cells in co-culture, leading to expensive and time-consuming methods for the production of transplantable cell populations. An alternative is to influence stem cell commitment with passive forces such as the presentation of specific microenvironmental factors through material characteristics.

Surface hydrophobicity has been shown to impact on the differentiation potential of embryonic stem cells, through the control of the embryoid body size during culture.^37^ Farrukh et al. demonstrated the synergistic effect of hydrogel stiffness and presentation of IKVAV peptide motifs, driving neuronal differentiation of embryonic and adult progenitor cells.^38^ In a similar approach, we have shown control over NSCs, with a number of chemical characteristic factors playing a role in determining the biological response at the materials interface.

#### 3.2.3 Neuronal Population

The ability to increase the neuronal fraction *in vitro* is critical to producing better cell transplant populations for neurodegenerative disorders. The density of neurons on amine-functionalized surfaces was found to be very similar to laminin-coated surfaces, remaining relatively consistent within the standard deviation across the 7 days in culture (Figure 4). This finding is particularly relevant when considering that laminin is the “gold standard” for neuronal cultures *in vitro* but that a general drive toward the 3R’s and/or clinical application is driving toward the use of non-animal-derived material substrates.

The similarity of the non-wettable phenyl and methyl surfaces highlights the importance of surface hydrophobicity in determining cell interactions; however, a simple trend relating the density of neurons to the substrate was not found with respect to either wettability nor p*K*_a_ as has been shown with the other cell types.^39^ This suggests a complex process, likely impacted due to the mixture of cell types in this co-culture system. Cell–surface and cell–cell communication results in an elaborate conditioning process, wherein differentiating glia and neurons mediate their environment through the deposition of signaling proteins. Attempting to fit such a complicated system into one variable, such as surface wettability, has been discussed in the literature by Dubiel et al. as being impossible.^40^ The balance of contributing factors may be difficult to fully interrogate within this study, although it is clear that surface functionality is an effective lever on neural density. It is not clear if there are multiple response factors in play, although it is likely that the surfaces are affecting cell division and possibly preferential support of certain cell types; this is indicated by our results when comparing neuronal densities, neural population ratios, and spreading characteristics.

#### 3.2.4 Cell Morphology and Neurite Outgrowth

When dealing with neuronal cultures, the morphology of neurites is often considered as a good measure of population characteristics; longer neurites are considered to be better for neuronal connectivity due to the increased ability for communication/synapse formation of these cells in culture or during transplantation. Specific peptide epitopes derived from laminin are known to steer the elongation of neurites, most notably IKVAV.^41^ Our findings support this, with the longest neurites measured on laminin surfaces, as shown in Figure 5. Amine surfaces did however also demonstrate the potential to support neuron elongation, possibly through the electrostatic interaction between the surface and the membrane walls, else through the directed adsorption of laminin from culture media.

Initially CH_3_-terminated surfaces gave rise to the highest proportion of short neurites (<500 μm), suggesting the low level of the first interaction of neurons on this surface, which increased with increasing culture time. This is supported by the increasing ability of neurons to migrate over carboxyl-terminated surfaces, evidenced by decreasing neuronal density (Figure 4). Neurospheres cultured on phenyl surfaces showed a similar trend, although no significant differences were observed between phenyl- and hydroxyl-presenting surfaces. This indicates that the initial cell interaction might steer early neurite outgrowth, with adsorbed proteins from media/secreted from cells during adhesion acting to mediate later stage neurite outgrowth. On all the surfaces, neurites were found to increase in length over the 7 days in culture. Others have reported similar measures, with no definitive surface characteristic being primarily critical to the late stage neurite elongation; Liu^33^ and Nakajima^34^ showed neural guidance and tethering of neuronal signaling factors using amine-rich poly(ethylenimine)-tethered surfaces. The lengthening of neurites and migrational (extensional) guidance can be directed through an ECM protein interaction, being influenced by hydrophilic and positively charged (at physiological pH) amine functionalities. Although wettability is a well-understood characteristic, it is clear that the classification of chemistry presented within the depth of a few carbon bond lengths of the surface needs to be considered and reported for full consideration.

## 4 Conclusions

The ability of cells to respond to their local environment is of key importance when considering the design of biomaterials for optimum cell culture *in vitro* and *in vivo*. By understanding specific cell–substrate interactions, and how they might lead to specific responses, biological surface engineers hope to be able to strongly influence cells, from differentiation to directed morphological control with respect to neurosphere spreading and neurite lengthening. In our experiments, surface chemical functionality was shown to have a dramatic impact on NSC and daughter cell responses. Clear differences were observed between all the surfaces and the cell response metrics, with amine surfaces giving rise to a similar attachment, spreading, and differentiation capacity as that shown by laminin-conditioned surfaces. The study presented shows that a simple and cheap chemical modification to a material’s surface controls various aspects of cell response, being of major benefit in terms of the 3Rs and scale-up, scale out of cellular therapies for the neural tissue.

## 5 Materials and Methods

### 5.1 Surface Fabrication

Glass coverslips (13 mm diameter, Thermo Scientific) were cleaned in a piranha etch solution using a 3:1 ratio of sulfuric acid and hydrogen peroxide (Fisher Scientific) and rinsed in deionized water and isopropanol (Fisher Scientific) before being air dried. These were then immersed directly in 1% vol/vol toluene–silane solutions for 24 h at room temperature, before being rinsed in toluene and air-dried. Samples were prepared immediately prior to use, being stored no longer than 2 days in a desiccator. Silanes used were all purchased from Sigma and used as received: tetraethyl orthosilicate (OH), (3-aminopropyl)trimethoxysilane (NH_2_), (3-mercaptopropyl)trimethoxysilane (SH), triethoxyphenylsilane (Ph), and chlorotrimethylsilane (CH_3_). Carboxyl surfaces were fabricated in a two-step process, reacting succinic anhydride (10% excess, Sigma) overnight at room temperature with pre-formed amine SAMs. Poly-d-lysine (PDL) laminin surfaces were prepared as standardized controls by incubation overnight at 4 °C; first in the poly-d-lysine solution (10 μg mL^–1^ in phosphate buffered saline, pH 7.4, 200 mmol salts; PBS, Sigma-Aldrich), followed by laminin solution (5 μg mL^–1^ in PBS), being rinsed thoroughly after each incubation with PBS, and then being air-dried. This was carried out to produce samples immediately prior to use.

### 5.2 Surface Analysis

Water contact angle measurements were made by placing a ∼5 μL drop of deionized water *via* a syringe with the immediate capture of the droplet image using Measurements & Automation software (National Instruments Corp. Austin, USA). Images were analyzed using ImageJ with the LB-ADSA plugin (EPFL), with a minimum of three droplets (six angles) being recorded per sample. X-ray photoelectron spectroscopy was performed at the Nexus NanoLAB (Newcastle University, UK) using a Theta Probe instrument equipped with a monochromated AlKα source (Thermo Scientific). A pass energy of 200 eV and a step size of 1.0 eV was employed for all survey spectra, while a pass energy of 40 eV and a step size of 0.1 eV were used for high-resolution spectra of the elements of interest. A flood gun was used for charge compensation. Data acquired were analyzed using CasaXPS software. p*K*_a_ and log *P* of the surface presented molecules were calculated from structural information using ACDlabs software v12.

### 5.3 Derivation of NSCs

Approval was given for all procedures from the Home Office and all experiments were conducted in accordance with the animal handling guidelines of Keele University. Developing midbrains were dissected out from embryonic day E12 Sprague-Dawley rat embryos. Time of mating was defined as E0. The tissue was digested using 0.1% trypsin in DMEM medium (Worthington Biomedical Corp. Reading) for 30 min at 37 °C. Following sedimentation, cells were pelleted *via* centrifugation (750 rpm, 3 min) and washed three times with 200 μL of 0.05% DNase (Worthington Biomedical Corp. Reading) containing medium. On the third wash, the pellet was centrifuged at 700 rpm for 5 min and dissociated to produce single cells. These were plated in a T25 in serum-free medium containing bFGF (10 ngmL^–1^ concentration).^16^ Neurospheres were cultured for 1 week to expand cell numbers.^17^ Dissociation after 3 days allowed cell expansion and control of heterogeneity by limiting the size range of any maturing neurospheres.^18^

### 5.4 Culture Conditions

SAM-coated coverslips were placed in a 24 well-plate format, each being seeded with whole neurospheres ensuring a pure mixture of NSCs, glia progenitors, and neural progenitors. Neurospheres were seeded directly onto SAM-modified glass coverslips and cultured for a further 3, 5, or 7 days, with paraformaldehyde fixation and staining carried out at each time point. PDL–laminin was used in the study as a reference to the current “gold-standard” surface used to support neural cultures *in vitro*. All the substrates were seeded with a microculture of ∼200 neurospheres in 30 μL of serum-rich (10%) culture media. These were left for 12 h before the addition of 0.5 mL culture media. Samples were cultured at 37 °C at 5% CO_2_, being fixed at three time points (3, 5, and 7 days) using 4% paraformaldehyde in phosphate-buffered saline. Three sample repeats were constructed from three separate tissue preparations (*n* = 9 total).

### 5.5 Immunohistochemistry

Fixed cells were stained for β_3_-tubulin (1:500) and GFAP (1:1000) using secondary antibodies tagged with fluorescent markers (Alexa Fluor 488 and 555, 1:300); all antibodies and markers were obtained from Abcam. Samples were mounted in DAPI-containing mounting media (Vector Labs, Peterborough). Samples were imaged on a Nikon epifluorescence microscope fitted with an auto-x-y scanning stage, allowing large-area stitched image acquisition. All images were taken at ×20 magnification. Confocal imaging was carried out on a Zeiss LSM 710 confocal laser scanning microscope fitted with 20× objective. Layer slices were captured at a resolution of 45 μm with successive layers being pictured.

### 5.6 Image Analysis

The quantification of image data was carried out using either Nikon NIS Elements or ImageJ software. Neurospheres were imaged (where possible) to cover the entire spreading area, with their boundary being assessed in terms of the edge cells staining positive for either GFAP or β_3_-tubulin. Numerical data were exported into Excel and OriginLab v8.5 (OriginLab Corporation, Massachusetts) for all statistical analysis. Two-way ANOVA and Tukey’s *post hoc* tests were used to understand statistical variance.

## Supporting Information Available

Images of water droplets resting on defined silane monolayers presenting differing terminal groups and XPS data for all surfaces (PDF)

## References

[ref1] Doulgkeroglou M.-N.; Di Nubila A.; Niessing B.; König N.; Schmitt R. H.; Damen J.; Szilvassy S. J.; Chang W.; Csontos L.; Louis S.; et al. Automation, Monitoring, and Standardization of Cell Product Manufacturing. Front. Bioeng. Biotechnol. 2020, 8, 811 10.3389/fbioe.2020.00811. 32766229 PMC7381146

[ref2] Roach P.; Parker T.; Gadegaard N.; Alexander M. R. A Bio-Inspired Neural Environment to Control Neurons Comprising Radial Glia, Substrate Chemistry and Topography. Biomater. Sci. 2013, 1, 83 10.1039/c2bm00060a. 32481998

[ref3] Roach P.; Parker T.; Gadegaard N.; Alexander M. R. Surface Strategies for Control of Neuronal Cell Adhesion: A Review. Surf. Sci. Rep. 2010, 65, 145–173. 10.1016/j.surfrep.2010.07.001.

[ref4] Richbourg N. R.; Peppas N. A.; Sikavitsas V. I. Tuning the Biomimetic Behavior of Scaffolds for Regenerative Medicine through Surface Modifications. J. Tissue Eng. Regener. Med. 2019, 13, 1275–1293. 10.1002/term.2859. PMC6715496 30946537

[ref5] Kriks S.; Shim J.-W.; Piao J.; Ganat Y. M.; Wakeman D. R.; Xie Z.; Carrillo-Reid L.; Auyeung G.; Antonacci C.; Buch A.; et al. Dopamine Neurons Derived from Human ES Cells Efficiently Engraft in Animal Models of Parkinson’s Disease. Nature 2011, 480, 547–551. 10.1038/nature10648. 22056989 PMC3245796

[ref6] Letourneau P. C.; Condic M. L.; Snow D. M. Interactions of Developing Neurons with the Extracellular Matrix. J. Neurosci. 1994, 14, 915 10.1523/jneurosci.14-03-00915.1994. 8120634 PMC6577522

[ref7] Konagaya S.; Kato K.; Nakaji-Hirabayashi T.; Iwata H. Design of Culture Substrates for Large-Scale Expansion of Neural Stem Cells. Biomaterials 2011, 32, 992–1001. 10.1016/j.biomaterials.2010.10.008. 21071075

[ref8] Piedade A. P.; Veneza C.; Duarte C. B. Polyamide 6.6 Thin Films with Distinct Ratios of the Main Chemical Groups: Influence in the Primary Neuronal Cell Culture. Appl. Surf. Sci. 2019, 490, 30–37. 10.1016/j.apsusc.2019.06.066.

[ref9] Rawsterne R. E.; Todd S. J.; Gough J. E.; Farrar D.; Rutten F. J. M.; Alexander M. R.; Ulijn R. V. Cell Spreading Correlates with Calculated LogP of Amino Acid-Modified Surfaces. Acta Biomater. 2007, 3, 715–721. 10.1016/j.actbio.2007.02.006. 17448740

[ref10] Patel A. K.; Tibbitt M. W.; Celiz A. D.; Davies M. C.; Langer R.; Denning C.; Alexander M. R.; Anderson D. G. High Throughput Screening for Discovery of Materials That Control Stem Cell Fate. Curr. Opin. Solid State Mater. Sci. 2016, 20, 202–211. 10.1016/j.cossms.2016.02.002.

[ref11] Ndyabawe K.; Cipriano M.; Zhao W.; Haidekker M.; Yao K.; Mao L.; Kisaalita W. S. Brain-on-a-Chip Device for Modeling Multiregional Networks. ACS Biomater. Sci. Eng. 2021, 7, 350–359. 10.1021/acsbiomaterials.0c00895. 33320530

[ref12] Hsu C.-H.; Huang T.-Y.; Chen R.-D.; Liu Y.-X.; Chin T.-Y.; Chen-Yang Y. W.; Yeh J.-M. Biomolding Technique to Fabricate the Hierarchical Topographical Scaffold of POMA To Enhance the Differentiation of Neural Stem Cells. ACS Biomater. Sci. Eng. 2017, 3, 1527 10.1021/acsbiomaterials.7b00091. 33429639

[ref13] Ren Y.-J.; Zhang H.; Huang H.; Wang X.-M.; Zhou Z.-Y.; Cui F.-Z.; An Y.-H. In Vitro Behavior of Neural Stem Cells in Response to Different Chemical Functional Groups. Biomaterials 2009, 30, 1036–1044. 10.1016/j.biomaterials.2008.10.028. 19026444

[ref14] Hung H.-S.; Yu A. Y.-H.; Hsieh S.-C.; Kung M.-L.; Huang H.-Y.; Fu R.-H.; Yeh C.-A.; Hsu S.-h. Enhanced Biocompatibility and Differentiation Capacity of Mesenchymal Stem Cells on Poly(Dimethylsiloxane) by Topographically Patterned Dopamine. ACS Appl. Mater. Interfaces 2020, 12, 44393–44406. 10.1021/acsami.0c05747. 32697572

[ref15] Jacques T. S.; Relvas J. B.; Nishimura S.; Pytela R.; Edwards G. M.; Streuli C. H.; ffrench-Constant C. Neural Precursor Cell Chain Migration and Division Are Regulated through Different Beta1 Integrins. Development 1998, 125, 3167–3177. 10.1242/dev.125.16.3167. 9671589

[ref16] Valamehr B.; Jonas S. J.; Polleux J.; Qiao R.; Guo S.; Gschweng E. H.; Stiles B.; Kam K.; Luo T.-J. M.; Witte O. N.; et al. Hydrophobic Surfaces for Enhanced Differentiation of Embryonic Stem Cell-Derived Embryoid Bodies. Proc. Natl. Acad. Sci. U.S.A. 2008, 105, 14459–14464. 10.1073/pnas.0807235105. 18791068 PMC2567159

[ref17] Silva G. A.; Czeisler C.; Niece K. L.; Beniash E.; Harrington D. A.; Kessler J. A.; Stupp S. I. Selective Differentiation of Neural Progenitor Cells by High-Epitope Density Nanofibers. Science 2004, 303, 1352–1355. 10.1126/science.1093783. 14739465

[ref18] Suslov O. N.; Kukekov V. G.; Ignatova T. N.; Steindler D. A. Neural Stem Cell Heterogeneity Demonstrated by Molecular Phenotyping of Clonal Neurospheres. Proc. Natl. Acad. Sci. U.S.A. 2002, 99, 14506 10.1073/pnas.212525299. 12381788 PMC137913

[ref19] Bain C. D.; Whitesides G. M. Modeling Organic Surfaces with Self-Assembled Monolayers. Angew. Chem. 1989, 101, 522–528. 10.1002/ange.19891010446.

[ref20] Khot G.; Kuforiji F.; Wright R.; Roach P. Dynamic Assessment of Fibrinogen Adsorption and Secondary Structure Perturbation. Conf. Pap. Sci. 2014, 2014, 601546 10.1155/2014/601546.

[ref21] Jenkins S. I.; Weinberg D.; Al-Shakli A. F.; Fernandes A. R.; Yiu H. H. P.; Telling N. D.; Roach P.; Chari D. M. “Stealth” Nanoparticles Evade Neural Immune Cells but Also Evade Major Brain Cell Populations: Implications for PEG-Based Neurotherapeutics. J. Controlled Release 2016, 224, 136 10.1016/j.jconrel.2016.01.013. 26780172

[ref22] Hung C.-H.; Young T.-H. Differences in the Effect on Neural Stem Cells of Fetal Bovine Serum in Substrate-Coated and Soluble Form. Biomaterials 2006, 27, 5901–5908. 10.1016/j.biomaterials.2006.08.009. 16945412

[ref23] Takahashi J.; Palmer T. D.; Gage F. H. Retinoic acid and neurotrophins collaborate to regulate neurogenesis in adult-derived neural stem cell cultures. J. Neurobiol. 1999, 38, 65 10.1002/(SICI)1097-4695(199901)38:1<65::AID-NEU5>3.0.CO;2-Q. 10027563

[ref24] Kokuzawa J.; Yoshimura S.; Kitajima H.; Shinoda J.; Kaku Y.; Iwama T.; Morishita R.; Shimazaki T.; Okano H.; Kunisada T.; et al. Hepatocyte Growth Factor Promotes Proliferation and Neuronal Differentiation of Neural Stem Cells from Mouse Embryos. Mol. Cell. Neurosci. 2003, 24, 190–197. 10.1016/S1044-7431(03)00160-X. 14550779

[ref25] Morgenthaler M.; Schweizer E.; Hoffmann-Röder A.; Benini F.; Martin R. E.; Jaeschke G.; Wagner B.; Fischer H.; Bendels S.; Zimmerli D.; et al. Predicting and Tuning Physicochemical Properties in Lead Optimization: Amine Basicities. ChemMedChem 2007, 2, 1100–1115. 10.1002/cmdc.200700059. 17530727

[ref26] Bernasconi C. F.; Leyes A. E.; Dötz K. H.; Fischer H.; Hofmann P.; Kreissl F. R.; Schubert U.; Weiss K.; Flores F. X.; Gandler J. P.; Transitional Metal Complexes; Verlag Chemie, 1997; Vol. 11.

[ref27] Smith M. B. March’s Advanced Organic Chemistry: Reactions, Mechanisms, and Structure, 8th ed.; Wiley-VCH Verlag, 2019.

[ref28] Curran J. M.; Chen R.; Hunt J. A. Controlling the Phenotype and Function of Mesenchymal Stem Cells in Vitro by Adhesion to Silane-Modified Clean Glass Surfaces. Biomaterials 2005, 26, 7057–7067. 10.1016/j.biomaterials.2005.05.008. 16023712

[ref29] Campos L. S.; Leone D. P.; Relvas J. B.; Brakebusch C.; Fässler R.; Suter U.; Ffrench-Constant C. β1 Integrins Activate a MAPK Signalling Pathway in Neural Stem Cells That Contributes to Their Maintenance. Development 2004, 131, 3433–3444. 10.1242/dev.01199. 15226259

[ref30] Fuentealba L. C.; Obernier K.; Alvarez-Buylla A. Adult Neural Stem Cells Bridge Their Niche. Cell Stem Cell 2012, 10, 698–708. 10.1016/j.stem.2012.05.012. 22704510 PMC3726005

[ref31] Lois C.; García-Verdugo J.-M.; Alvarez-Buylla A. Chain Migration of Neuronal Precursors. Science 1996, 271, 978–981. 10.1126/science.271.5251.978. 8584933

[ref32] Bain C. D.; Whitesides G. M. Depth Sensitivity of Wetting: Monolayers of ω-Mercapto Ethers on Gold. J. Am. Chem. Soc. 1988, 110, 5897–5898. 10.1021/ja00225a050.

[ref33] Liu B. F.; Ma J.; Xu Q. Y.; Cui F. Z. Regulation of Charged Groups and Laminin Patterns for Selective Neuronal Adhesion. Colloids Surf., B 2006, 53, 175–178. 10.1016/j.colsurfb.2006.08.018. 17046215

[ref34] Nakajima M.; Ishimuro T.; Kato K.; Ko I.-K.; Hirata I.; Arima Y.; Iwata H. Combinatorial Protein Display for the Cell-Based Screening of Biomaterials That Direct Neural Stem Cell Differentiation. Biomaterials 2007, 28, 1048–1060. 10.1016/j.biomaterials.2006.10.004. 17081602

[ref35] Grandbarbe L.; Bouissac J.; Rand M.; Hrabé de Angelis M.; Artavanis-Tsakonas S.; Mohier E. Delta-Notch Signaling Controls the Generation of Neurons/Glia from Neural Stem Cells in a Stepwise Process. Development 2003, 130, 1391–1402. 10.1242/dev.00374. 12588854

[ref36] Chen E.; Xu D.; Lan X.; Jia B.; Sun L.; Zheng J.; Peng H. A Novel Role of the STAT3 Pathway in Brain Inflammation-Induced Human Neural Progenitor Cell Differentiation. Curr. Mol. Med. 2013, 13, 1474–1484. 10.2174/15665240113139990076. 23971732 PMC4157724

[ref37] Valamehr B.; Jonas S. J.; Polleux J.; Qiao R.; Guo S.; Gschweng E. H.; Stiles B.; Kam K.; Luo T.-J. M.; Witte O. N.; et al. Hydrophobic Surfaces for Enhanced Differentiation of Embryonic Stem Cell-Derived Embryoid Bodies. Proc. Natl. Acad. Sci. U.S.A. 2008, 105, 14459–14464. 10.1073/pnas.0807235105. 18791068 PMC2567159

[ref38] Farrukh A.; Ortega F.; Fan W.; Marichal N.; Paez J. I.; Berninger B.; Campo A. d.; Salierno M. J. Bifunctional Hydrogels Containing the Laminin Motif IKVAV Promote Neurogenesis. Stem Cell Rep. 2017, 9, 1432–1440. 10.1016/j.stemcr.2017.09.002. PMC5829305 28988991

[ref39] Mei Y.; Saha K.; Bogatyrev S. R.; Yang J.; Hook A. L.; Kalcioglu Z. I.; Cho S.-W.; Mitalipova M.; Pyzocha N.; Rojas F.; et al. Combinatorial Development of Biomaterials for Clonal Growth of Human Pluripotent Stem Cells. Nat. Mater. 2010, 9, 768 10.1038/NMAT2812. 20729850 PMC3388774

[ref40] Dubiel E. A.; Martin Y.; Vermette P. Bridging the Gap between Physicochemistry and Interpretation Prevalent in Cell-Surface Interactions. Chem. Rev. 2011, 111, 2900–2936. 10.1021/cr9002598. 21319750

[ref41] Jain R.; Roy S. Controlling Neuronal Cell Growth through Composite Laminin Supramolecular Hydrogels. ACS Biomater. Sci. Eng. 2020, 6, 2832–2846. 10.1021/acsbiomaterials.9b01998. 33463249

