# Supplementary material for: Effects of Surface Chemistry Interaction on Primary Neural Stem Cell Neurosphere Responses
Source: ACS Omega. 2021 Jul 19;6(30):19901–10. doi: 10.1021/acsomega.1c02796 (PMC8340405; doi:10.1021/acsomega.1c02796)
Supplement: Supplementary file 1 — ao1c02796_si_001.pdf [file ao1c02796_si_001.pdf]

Effects of Surface Chemistry Interaction on Primary Neural Stem Cell Neurosphere Responses

Georghios Joseph[1], Rowan P. Orme[1], Theocharis Kyriacou[2], Rosemary A. Fricker[1] and Paul Roach[3]*

[1] Institute for Science and Technology in Medicine, and School of Medicine, Keele University, Keele, Staffs, ST5 5BG UK

[2] School of Computing and Mathematics, Keele University, Keele, Staffs, ST5 5BG UK

[3] Department of Chemistry, School of Science, Loughborough University, Loughborough, Leicestershire. LE11 3TU. UK

The following figures are given in support to the main text.

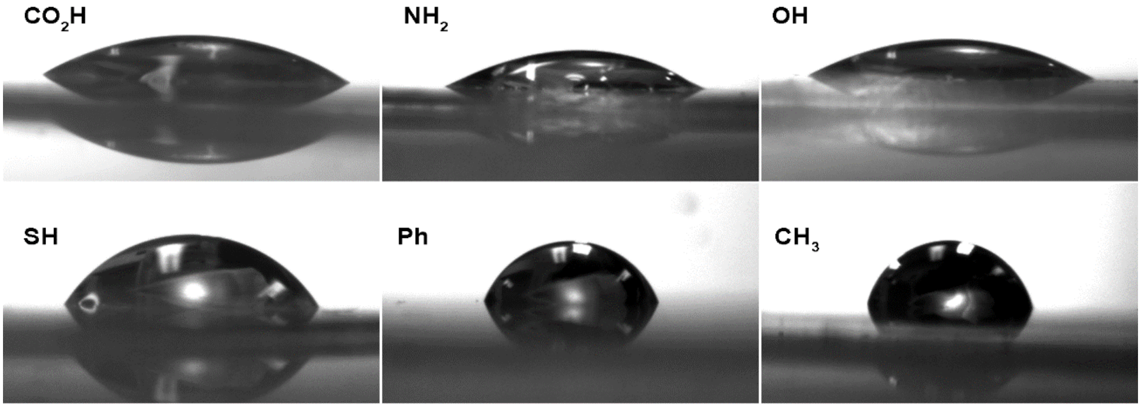

Figure S1. Water contact angle images showing droplets on prepared self-assembled monolayers.

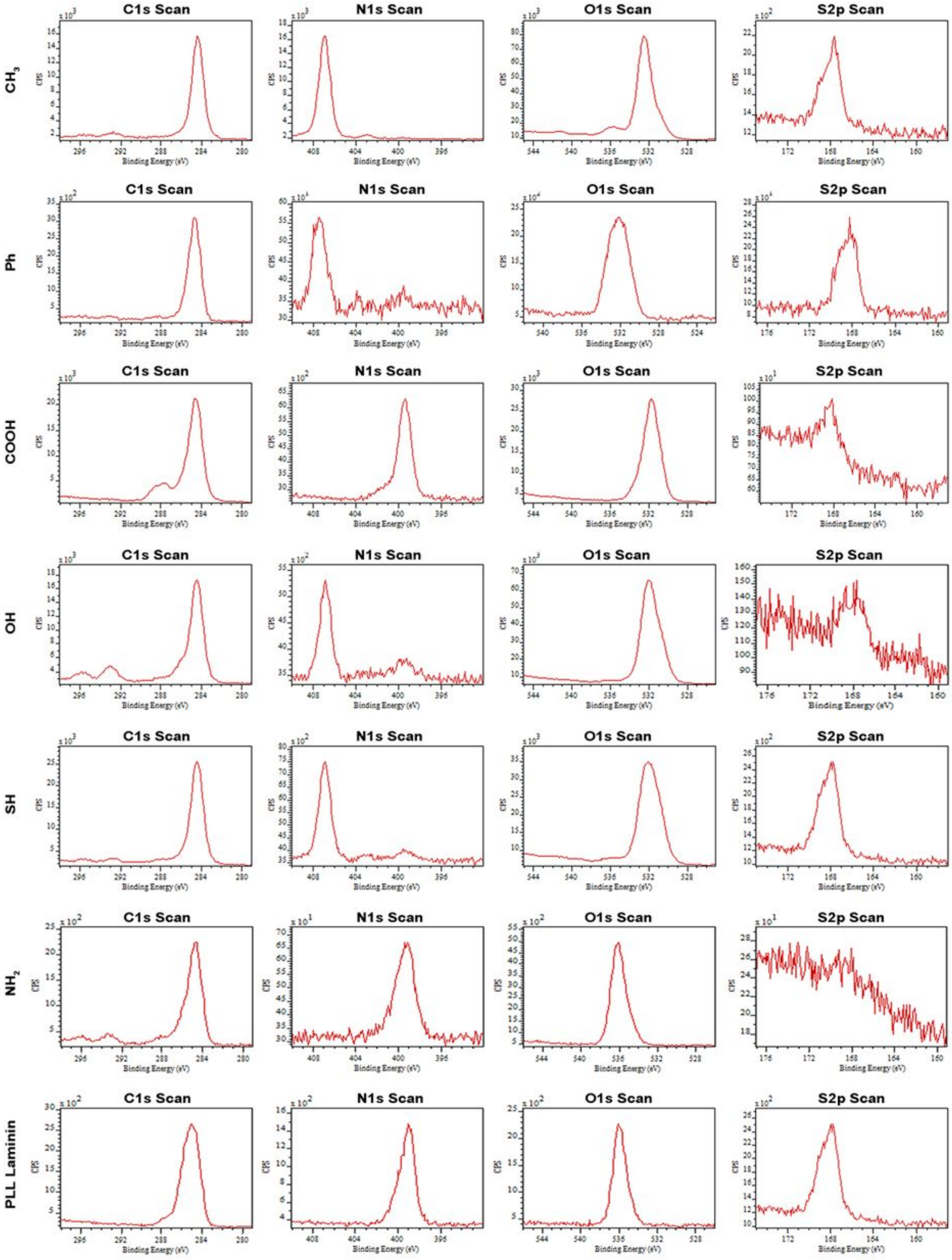

Figure S2. X-ray photoelectron spectroscopy of self-assembled monolayers.
